# Variability of the mineral composition of durum wheat grain (Triticum durum Desf.) under different environmental conditions

**DOI:** 10.18699/vjgb-25-86

**Published:** 2025-10

**Authors:** I.N. Leonova, P.N. Malchikov, N.А. Vinichenko, V.V. Piskarev, M.G. Myasnikova, V.А. Aparina, Т.V. Chaheeva

**Affiliations:** Institute of Cytology and Genetics of the Siberian Branch of the Russian Academy of Sciences, Novosibirsk, Russia; Institute of Cytology and Genetics of the Siberian Branch of the Russian Academy of Sciences, Novosibirsk, Russia Samara Federal Research Scientific Center of the Russian Academy of Sciences, Samara Scientific Research Agriculture Institute named after N.M. Tulaykov, Bezenchuk, Samara region, Russia; Institute of Cytology and Genetics of the Siberian Branch of the Russian Academy of Sciences, Novosibirsk, Russia; Institute of Cytology and Genetics of the Siberian Branch of the Russian Academy of Sciences, Novosibirsk, Russia Siberian Research Institute of Plant Production and Breeding – Branch of the Institute of Cytology and Genetics of the Siberian Branch of the Russian Academy of Sciences, Krasnoobsk, Novosibirsk region, Russia; Samara Federal Research Scientific Center of the Russian Academy of Sciences, Samara Scientific Research Agriculture Institute named after N.M. Tulaykov, Bezenchuk, Samara region, Russia; Institute of Cytology and Genetics of the Siberian Branch of the Russian Academy of Sciences, Novosibirsk, Russia Siberian Research Institute of Plant Production and Breeding – Branch of the Institute of Cytology and Genetics of the Siberian Branch of the Russian Academy of Sciences, Krasnoobsk, Novosibirsk region, Russia; Samara Federal Research Scientific Center of the Russian Academy of Sciences, Samara Scientific Research Agriculture Institute named after N.M. Tulaykov, Bezenchuk, Samara region, Russia

**Keywords:** durum wheat, macroelements, microelements, heavy metals, твердая пшеница, макроэлементы, микроэлементы, тяжелые металлы

## Abstract

The composition of wheat grain plays a key role in determining its nutritional value. In this work, a collection of 133 durum wheat varieties and breeding lines was assessed for the content of macroelements (Ca, Mg, K), microelements (Cu, Mn, Zn, Fe, Na) and toxic metals (Pb, Cd, and Cr) in grain under the environmental conditions of Samara and Novosibirsk regions in 2023. The results showed a wide range of variations in the concentration of all elements depending on genotypic differences between the samples as well as the growing region. Ca and Mg contents in the varieties grown in Samara region showed a significant excess of 3.1- and 1.5-fold, respectively. Zn, Pb, and Cr content in the varieties cultivated in Novosibirsk turned out to be two times as high. Statistical analysis of element concentrations in the varieties of different origin indicates that Russian breeding lines significantly outperform Russian cultivars in Mg content, while being inferior in K, Cu, and Mn. Compared to Russian cultivars and breeding lines, foreign varieties demonstrated higher contents of K and heavy metals Cd and Cr. Correlation analysis using mean values of indicators for two environments showed highly significant ( p < 0.001) positive relationships between the content of microelements Fe/Mn (r2 = 0.69), Fe/Zn (r2 = 0.49), and Zn/Mn (r2 = 0.46), which suggests a feasibility of selecting genotypes for several elements at once. Multivariate statistics divided the durum wheat collection into two groups, one of them including Russian cultivars and breeding lines as well as some foreign genotypes. A separate cluster included seven Russian breeding lines placed at a distance from the other varieties, which suggested their potential differences at the genetic level. Comparing these lines with respect to mineral composition showed that they were, on average, characterized by higher Mg, K, Zn, and Fe contents. The data obtained in this study can be used for genetic research and breeding to improve the grain mineral composition of the modern durum wheat varieties

## Abstract (ru)

Минеральный состав зерна пшеницы играет ключевую роль в определении его питательной цен- ности. В данной работе проведена оценка коллекции из 133 сортов и селекционных линий яровой твердой пшеницы отечественного и иностранного происхождения по содержанию в зерне макроэлементов (Ca, Mg, K), микроэлементов (Cu, Mn, Zn, Fe, Na) и токсичных металлов (Pb, Cd, Cr) при выращивании в экологических условиях Самарской и Новосибирской областей. Результаты показали широкий размах варьирования концен- трации всех элементов в зависимости от генотипических различий между образцами и региона выращивания. Значительное превышение концентрации Ca и Mg в 3.1 и 1.5 раза соответственно отмечено у сортообразцов, выращенных в Самарской области. Культивирование образцов в условиях Новосибирской области сопровождалось превышением содержания Zn, Pb и Cr в зерне более чем в два раза. Статистический анализ содержания минеральных элементов у образцов различного происхождения свидетельствует о том, что российские селек- ционные линии достоверно превышают отечественные сорта по содержанию Mg, но при этом уступают по кон- центрации К, Cu и Mn. Сортообразцы иностранной селекции отличались от российских сортов и линий повы- шенным содержанием K и тяжелых металлов Cd и Cr. Анализ корреляций, проведенный на основании средних значений показателей по двум регионам, указывает на наличие высокодостоверных (p < 0.001) положительных взаимосвязей между содержанием микроэлементов Fe/Mn (r2 = 0.69), Fe/Zn (r2 = 0.49) и Zn/Mn (r2 = 0.46), что предполагает возможность проведения отбора генотипов по нескольким элементам одновременно. Много- мерный статистический анализ разделил образцы на две группы, одна из которых включала российские сорта и селекционные линии, а также часть иностранных образцов. Отдельный кластер состоял из семи российских селекционных линий, расположенных дистантно от остальных образцов, что предполагает их различия на ге- нетическом уровне. Сравнение этих линий по минеральному составу показало, что линии в среднем характе- ризуются более высокими концентрациями Mg, K, Zn и Fe. Данные по содержанию микро- и макроэлементов в изученных сортообразцах яровой твердой пшеницы могут быть использованы для генетических исследова- ний и практической селекции для улучшения существующих сортов по минеральному составу.

## Introduction

Mineral micro- and macronutrients play a significant role in maintaining human body functions and have a substantial impact on human health. The functional role of most mineral elements is diverse. In particular, they act as cofactors in various enzymatic reactions and are also involved in redox reactions during electron transfer, oxygen binding and transport in tissues, interaction of molecules with a cellular receptor, and regulation of gene expression (Sigel et al., 2013; Jomova et al., 2022; Islam et al., 2023).

Despite the importance of mineral nutrients, their optimal doses are also critical for the normal functioning of the body, as both excess and deficiency of certain minerals can lead to various physiological disorders. Deficiency of macroelements such as calcium, magnesium, and potassium causes muscular system disorders, changes in hormonal status, and may lead to malignant growths (Zoroddu et al., 2019; Ali, 2023). Iron deficiency is among the causes of anemia, cardiovascular diseases, and immune system disorders due to iron being incorporated into hemoproteins, such as hemoglobin and enzymes for xenobiotic degradation (Camaschella, 2019; Dixit et al., 2020). Insufficient zinc intake leads to growth and sexual development delays, reduced immunity, and various mental disorders (Hambidge, 2000). Symptoms of copper deficiency may include various joint lesions and pigmentation disorders in skin and hair (Olivares, Uauy, 1996). On the other hand, excessive intake of zinc, iron, and copper can lead to liver fibrosis and cirrhosis, neurodegenerative diseases, impaired immune and cognitive functions, and severe forms of anemia (Wessling-Resnick, 2017; Schoofs et al., 2024).

In addition to vital micro- and macroelements required in optimal concentrations, humans are also exposed to a number of toxic metals (lead, cadmium, mercury, chromium, aluminum), which can enter the body through food and have negative effects. The main toxicity mechanisms of heavy metals include mitochondrial apoptosis, interference with various signaling pathways and oxidative stress, changes in gene activity regulation due to various types of DNA damage, all of which can lead to the development of chronic diseases and the emergence of malignancies (Kiran et al., 2022; Jomova et al., 2025).Food products are the main sources of minerals for the human body. For most of the world’s population, food products made from bread and durum wheat are the main sources of protein, vitamins, and minerals. Bread wheat (Triticum aestivum L., 2n = 42, AABBDD genome) is one of the most valuable food grain crops, ranking among the highest-rated crops in most countries. Daily consumption of bread wheat products provides up to 20 % of the required calories and up to 10–15 % of iron and zinc (Tadesse et al., 2019; Aghalari et al., 2022).

Unlike bread wheat, durum wheat (Triticum durum Desf., 2n = 28, AABB genome) is the only raw material for the production of high-quality macaroni products with a characteristic amber color and excellent taste. According to available data, a significant number of cultivated durum wheat varieties outperform bread wheat varieties in the content of zinc, iron, calcium, magnesium, and other minerals (Cakmak et al., 2010; Del Coco et al., 2019; Saini et al., 2023). The main producers and consumers of durum wheat products are the Mediterranean countries (Italy, Turkey, Greece, Tunisia, France), which account for more than 50 % of the cultivated area. Other major suppliers of durum wheat include Canada, the United States, Mexico, India, and Kazakhstan. Until the mid-20th century, Russia was one of the major producers of durum wheat grain and ranked first in the world in terms of sown area, which reached 20 million hectares (Martínez-Moreno et al., 2022; Malchikov, Myasnikova, 2023). A significant reduction in sowing and grain harvesting occurred in the early 1990s after the collapse of the USSR, and until recently, the area under durum wheat was estimated at ~0.7 million hectares, which is no higher than 1.7 % of the global area (Goncharov, Kurashov, 2018).

In recent years, considerable attention has been paid to improving the mineral composition of wheat grain and increasing the concentration of essential nutrients. Biofortification breeding programs were used as vehicles to introduce the newly developed bread wheat samples with genetically increased zinc and iron contents (Khokhar et al., 2018; Virk et al., 2021; Tanin et al., 2024). Efforts on developing biofortified breeding lines of bread wheat and searching for donors with high protein, mineral, and antioxidant content are made in the Russian Federation as well (Morgounov et al., 2022; Potapova et al., 2023; Gordeeva et al., 2024). The data from long-term trials of wheat landraces and modern varieties as well as breeding and introgression lines focusing on grain quality and mineral composition traits have been systematized, which made it possible to identify the genotypes with target traits for breeding schemes (Shepelev et al., 2022; Orlovskaya et al., 2023; Leonova et al., 2024; Shamanin et al., 2024). However, when it comes to durum wheat, similar studies on the genetic diversity of Russian varieties and breeding lines in terms of grain mineral composition are critically lacking (Pototskaya et al., 2023; Sochalova et al., 2023).

The goal of this study was to investigate the genetic variability in contents of microelements (zinc, iron, copper, and manganese), macroelements (calcium, magnesium, and potassium), and toxic metals (lead, cadmium, and chromium) in the collection of spring durum wheat varieties and breeding lines grown under the environmental conditions of the Samara and Novosibirsk regions.

## Materials and methods

Plant material and field trial conditions. Plant material consisted of 133 durum wheat samples, including 35 Russian cultivars, 68 Russian breeding lines, 29 foreign accessions, and Turanian wheat Khorasan (Table S1)1. Among the Russian breeding lines, 39 genotypes were developed at the Samara Scientific Research Agriculture Institute, whereas the remaining ones originated from other Russian breeding centers. The samples were grown under field trial conditions on the premises of research institutes in the Novosibirsk and Samara regions in 2023. In the Novosibirsk region, the sowing was carried out in the field of the Siberian Research Institute of Plant Production and Breeding – a branch of the Institute of Cytology and Genetics SB RAS (54°54ʹ51.4″ N, 82°58ʹ37.1″ E). The soils of the experimental site are mainly leached chernozem of medium depth. The humus content in the topsoil is 5.7–6.9 %. The soils are highly supplied with mobile soil phosphates and potassium. The contents per 100 g of soil are as follows: Р2О5 – 42 mg, K2O – 35 mg. The total nitrogen content in the soil before sowing was 0.31 %. Sowing was done manually in furrows at a depth of 5–7 cm. The plot size was 0.4 m2, in two replicates.


Supplementary Materials are available in the online version of the paper: https://vavilov.elpub.ru/jour/manager/files/Suppl_Leonova_Engl_29_6.xls


The experimental fields of the Samara Scientific Research Agriculture Institute – a branch of the Samara Federal Research Scientific Center of the Russian Academy of Sciences – are located in the urban-type settlement of Bezenchuk (52°05ʹ85.5″ N, 49°02ʹ55.9″ E). The main soil type is common chernozem with medium to heavy loam texture. The average humus content is 4.8 %, nitrogen – 5.9 mg/kg, phosphorus – 279 mg/kg, potassium — 203 mg/kg. The variety samples were sown in a randomized layout on plots of 7.4 m2 in two replicates. The variety Bezenchukskaya 210 was used as a standard in both fields.

The comparison of the climatic conditions during the growing season between the regions, as well as the comparison of the results with long-term average values showed that in 2023, Novosibirsk region experienced elevated air temperatures throughout the growing season, with the exception of the second ten-day periods of May and August, when temperatures were below the long-term average (–1.3 and –2.0 °C against the long-term average, respectively) (Table S2). The most significant increase (+8.4 ºC) above the long-term average temperature was recorded in the first ten days of June, while precipitation during this period was only 27.8 % of the long-term average. Overall, the first half of the growing season (May–June) was characterized by a severe lack of precipitation (14.9 and 47.5 %, respectively), while in July precipitation fell within the normal range (102.1 %), and in August it was significantly above the long-term average (167.6 %). In the Samara region, there was a general precipitation deficit throughout the growing season against a background of moderate temperatures. From seedling stage to wax ripeness, the total precipitation was 89.3 mm. The most favorable conditions for durum wheat cultivation during the growing season occurred in the first and third ten-day periods of June, during the booting and heading stages, respectively

Content evaluation of micro- and macronutrients and heavy metals. The grains of durum wheat samples were analyzed with respect to contents of eight micro- and macronutrients (zinc, iron, copper, manganese, sodium, calcium, magnesium, and potassium) and three toxic metals (lead, cadmium, and chromium). Here, a 300 mg of grain was treated with 1 ml of hydrogen peroxide (60 %) and 5 ml of concentrated nitric acid. Sample mineralization was carried out in a microwave oven for 40 minutes. The sample volume was then brought to 50 ml with deionized water and diluted 50 times for element quantification. The chemical composition was analyzed using an atomic absorption spectrometry (AAS) device ContrAA 800 D (Analytik Jena, Germany). Each sample was analyzed in duplicate.Statistical analysis. Statistical analysis was performed using the Statistica v. 10 software (StatSoft, Inc., USA). The significance of differences between mean trait values was assessed using the Mann–Whitney test and Student’s t-test. Trait values are presented as means (M) and standard deviations (± SD). To evaluate the effects of genotype and environmental factors, two-way analysis of variance (ANOVA) was used. The relationship between the contents of various elements was assessed using Spearman’s correlation coefficient. Principal component analysis (PCA) and dendrogram plotting were performed using the PAST v. 4.03 software (Hammer et al., 2001). The UPGMA (unweighted pair group method with arithmetic mean) method was used for dendrogram plotting, and statistical significance of clustering was evaluated using a permutation test with 1,000 iterations.

## Results

The assessment of mineral element content in the durum wheat grain grown under the environmental conditions of two regions (Samara and Novosibirsk regions) revealed a wide range of variation in nutrient concentration, as well as differences depending on the growing region (Table 1; Table S1). The most significant regional differences were observed for elements such as Ca and Mg, the content of which in field conditions of the Samara region was 3.1 and 1.5 times higher, respectively. For samples grown in the Novosibirsk region, higher concentrations of Zn, Pb, and Cr were recorded: specifically 2.5, 2.3, and 2.2 times higher, respectively. All regional differences in element contents were statistically significant (p < 0.0001), except for Na (Table 1), the concentration of which was the same in both regions. The distribution of most mineral contents in genotypes grown in both fields was approximately normal, with the exception of lead concentration in the field of the Samara Scientific Research Agriculture Institute, which was significantly shifted toward lower values.

**Table 1.**
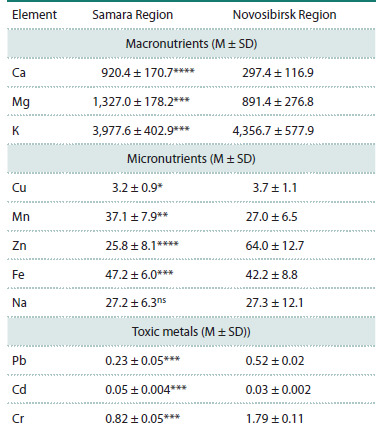
Contents of micro- and macronutrients and toxic metals (mg/kg) in grains of the durum wheat varieties grown under the environmental conditions of the Samara and Novosibirsk regions in 2023 Note. The data are presented as mean value (М) ± standard deviation (SD); * p < 0.05, ** p < 0.01, *** p < 0.001, **** p < 0.0001, ns – differences are nonsignificant.

ANOVA used to assess the effects of genotype and environmental factors on the phenotypic expression of traits showed that field conditions most affected the contents of Ca and Zn, with low and non-significant contribution of genotype observed for Ca concentration (Table S3). In contrast, Na content was mainly influenced by genotypic differences. A significant genotypic effect was found for the concentrations of Mg, K, Cu, Fe, and Cd, which substantially exceeded the influence of environmental factors

Since the studied collection consisted of several groups of varieties of different origin, it was of interest to determine whether these groups differed in element concentrations. It can be seen from Table 2 that Russian breeding lines significantly exceeded Russian cultivars in Mg content, but had lower levels of K, Cu, and Mn. Foreign varieties differed from Russian cultivars and lines by higher potassium content. The concentrations of toxic elements Cd and Cr were significantly higher on average in foreign samples compared to Russian ones, specifically 1.9 and 1.8 times higher, respectively. No significant differences between groups were found for Zn and Fe concentrations.

**Table 2.**
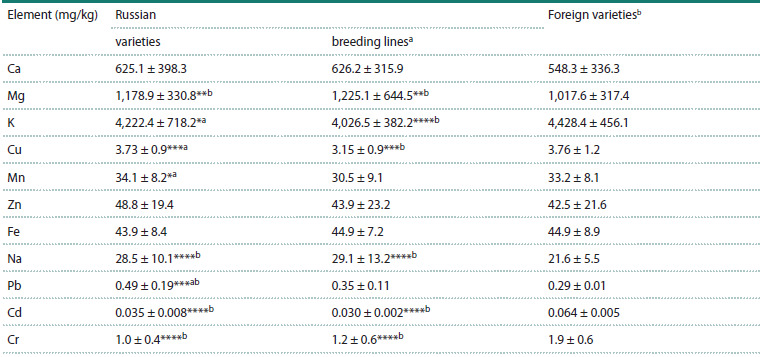
Contents of micro- and macronutrients and toxic metals in grains of varieties and breeding lines of Russian and foreign origin (Samara and Novosibirsk regions, 2023) Note. The data are presented as mean value ± standard deviation. Letters (a, b) indicate statistically significant differences between groups; * p <0.05, ** p < 0.01, *** p < 0.001, **** p < 0.0001.

According to the averaged data from both regions, the highest Ca content (>700 mg/kg) was observed in the Russian varieties Pamyati Chekhovicha, Zhemchuzhina Sibiri, Annushka, Krasnokutka 13, and in the Russian breeding lines L73, L75, and L76 developed at the Altai Research Institute of Agriculture. In terms of Mg content, the breeding lines L21, L23, L24, L25, and L26 from the Samara Scientific Research Agriculture Institute stood out, with element concentrations exceeding 2,000 mg/kg. The Russian varieties Annushka, Bezenchukskaya Stepnaya, Volnodonskaya, and the foreign varieties Hyperno and Tamaroi were characterized by high zinc and iron content (>54 and 51 mg/kg, respectively). High Cd concentrations, which in some cases exceeded permissible limits, were detected in the foreign varieties Tessadur, Achille, Fuego, and the breeding lines L51 and L56 (Table S1). The high levels of elements such as Mg (1,598.2 mg/kg), K (4,536.5 mg/kg), and Zn (56.1 mg/kg) observed in the ancient wheat Khorasan are also worth noting, confirming the data obtained by other authors and suggesting the potential of this species for improving the nutritional value of modern durum wheat varieties (Bordoni et al., 2017).

**Fig. 1.**
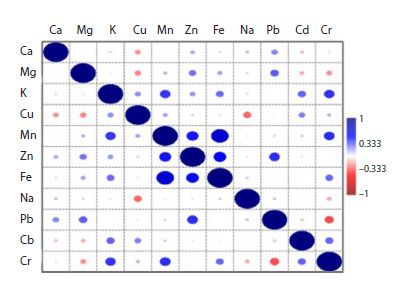
Genotypic correlations between the concentrations of micro- and macronutrients and toxic metals in grains of durum wheat cultivars and breeding lines.

Spearman correlation test based on the mean values from both regions indicated highly significant positive correlations between Fe and Mn (r2 = 0.69), Fe/Zn (r2 = 0.49), Zn/Mn (r2 = 0.46), Zn/Pb (r2 = 0.41), and Cr/K (r2 = 0.41) (Fig. 1; Table S4). Weak negative correlations were observed in the pairs as follows: Cu/Ca, Cu/Mg, Cu/Na, and Pb/Cr (r2 = −0.22, −0.24, −0.27, and −0.34, respectively).

**Fig. 2.**
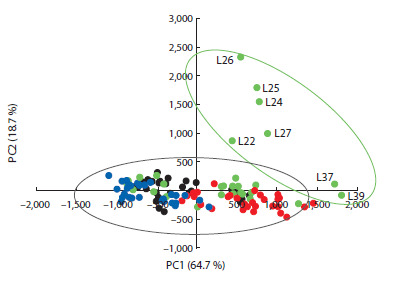
PCA plot illustrating the distribution of durum wheat genotypes in the space of the first two principal components Durum wheat samples are color-coded as follows: black – Russian varieties; blue – breeding lines from Russian institutions; green – breeding lines from the Samara Research Institute; red – foreign varieties

The results of mineral content assessment in the two regions were used to identify potential clustering of durum wheat varieties. Principal component analysis (PCA) was used to analyze the relationship between element concentrations and the affiliation of genotypes to the groups of different origin: Russian varieties, breeding lines from the Samara Scientific Research Agriculture Institute, breeding lines from other Russian breeding centers, and foreign accessions (Fig. 2).

Considering the first two principal components, which account for 64.7 and 18.7 % of the genetic variation, respectively, all studied varieties are divided into two groups. Most of the genotypes are clustered into one large group, with Russian durum wheat cultivars, breeding lines from Russian centers, and partially lines from the Samara Scientific Research Agriculture Institute grouped on the left side of this cluster. Foreign varieties are primarily located in the lower right part of the cluster. Seven lines (L22, L24, L25, L26, L27, L37, and L39) form a separate group and are positioned at a considerable distance from the other samples, which may indicate genetic-level differences. Comparing the mineral compositions of these lines showed that, on average, they were characterized by higher concentrations of Mg, K, Zn, and Fe (Table S1).

**Fig. 3.**
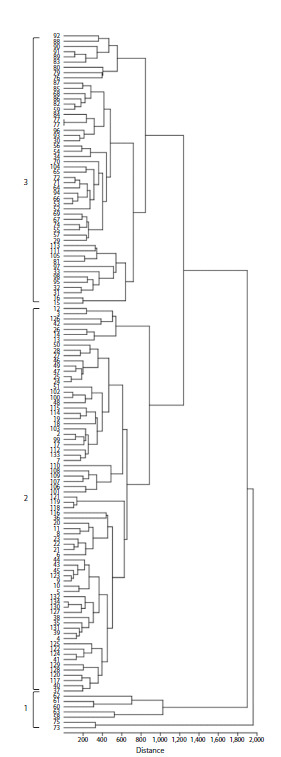
UPGMA dendrogram illustrating the clustering of durum wheat genotypes based on the assessment of concentrations of 11 micro- and macronutrients and toxic metals in grains.

Clustering of the samples using the UPGMA method confirmed the presence of two main groups (Fig. 3). Cluster 1 includes seven breeding lines (L22, L24, L25, L26, L27, L37, and L39) from the Samara Scientific Research Agriculture Institute and is clearly separated from the other samples. The second cluster is divided into two subclusters (labeled 2 and 3 in the Figure), one of which mainly includes Russian varieties and breeding lines from Russian breeding centers. Foreign varieties are predominantly grouped in the right part of subcluster 3.

## Discussion

In this paper, we investigated the grain concentrations of 11 micro- and macronutrients and toxic metals in durum wheat genotypes of both Russian and foreign origin, grown under the environmental conditions of the Samara and Novosibirsk regions. A wide range of variation was observed for all elements depending on both genotype and environmental factors. Significant regional differences were identified in the concentrations of essential macro- and micronutrients (Ca, K, and Zn) and toxic metals (Pb, Cd, and Cr) (Table S1). It is worth noting that the variation range for K, Mg, Cu, Mn, and Fe in both regions was comparable to the results reported by I.V. Pototskaya et al. (2023) obtained from the cultivation of 20 Russian and Kazakh durum wheat varieties in the Omsk region. An exception was observed for Ca and Zn contents, with the former being significantly higher and the latter lower in the Omsk fields compared to Novosibirsk.

An important aspect is the grain concentration of toxic metals, since exceeding the maximum permissible concentrations (MPCs) of essential trace nutrients such as Zn and Cu in food products can lead to harmful effects. Most varieties grown in the Novosibirsk region showed zinc concentrations in grains above the MPC (50 mg/kg), whereas the varieties from the Samara region showed contents falling within the acceptable range (Table S1). These differences may be related to zinc content in soils of the respective regions. Similarly, an increased lead content in grains was observed in nearly half of the genotypes grown in both regions.

According to a 30-year monitoring study of the mineral composition of soils in the chernozems of Western Siberia, no exceedance of the MPCs for mobile forms of heavy metals such as Cu, Zn, Pb, Cd, and Cr was detected (Krasnitsky et al., 2024). Soil analysis in the Samara region also indicates that the content of mobile forms of lead, nickel, chromium, copper, and zinc in all soil types and subtypes with varying composition and humus content does not exceed the MPCs (Obushchenko, Gnedenko, 2014). However, no analysis of toxic metals in the soils of the experimental fields was conducted, and this issue requires further investigation

Changes in heavy metal concentrations in soil may be influenced by factors such as soil preparation systems, application of organomineral fertilizers, use of pesticides and plant protection products, atmospheric precipitation, and various anthropogenic impacts from industrial enterprises, transportation, and others. Several studies have shown that systematic introduction of mineral and organic fertilizers contributes to the accumulation of heavy metals in soil and may affect the mineral composition and concentration in wheat grains (Ryan et al., 2004; Pandino et al., 2020; Wysocka et al., 2025). Root and foliar treatments with nitrogen fertilizers at various concentrations can increase the uptake and accumulation of Cd in durum wheat grains even at low soil Cd levels (Özkutlu, 2024). The introduction of organomineral fertilizers to barley crops in the soils of the Samara region, for example, led to an increase in the total content and mobility of heavy metals (Cu, Cd, Pb, Zn) and enhanced Pb migration into spring wheat plants (Trots V.B. et al., 2015; Trots N.M., Bokova, 2024).

Heavy metal contamination may also occur in soils located near industrial facilities (Prosyannikov, 2014). The mechanisms of heavy metal transfer and accumulation in soil–plant systems are highly complex, and the intensity of accumulation depends on the type of toxic element, weather conditions, regional soil structure, as well as the species and genotype of the plant. However, despite the increase in heavy metal concentrations in agricultural soils, most authors report that this does not significantly affect the accumulation of toxic substances in final agricultural products (Protasova, 2014; Wang et al., 2017; Ugulu et al., 2021).

The data obtained on the concentrations of micro- and macronutrients and heavy metals were used to study the grouping of genotypes in principal component space and to plot a phylogenetic tree. The results showed no clear separation into contrasting groups, except for a small number of lines developed at the Samara Scientific Research Agriculture Institute and foreign varieties (Fig. 2 and 3). Russian lines from different breeding centers formed a single cluster with durum wheat cultivars introduced into production in different years, which may indicate insufficient genetic diversity among both varieties and breeding lines. The modern global durum wheat pool is characterized by moderate genetic diversity, as confirmed by the results obtained from a wide range of genotypes from different countries (Zhao et al., 2009; Hakki et al., 2014; Hocaoğlu et al., 2020; Naseri et al., 2024).

The genetic diversity of Russian spring durum wheat varieties developed between 1929 and 2004 was previously assessed using pedigree data (Martynov et al., 2004). The analysis revealed that about 20 % of the pool of original Russian durum wheat varieties previously used in hybridization had been lost. A similar conclusion can be drawn for foreign durum wheat varieties, as breeding efforts over a long period focused primarily on developing high-yielding genotypes with resistance to diseases and lodging, which led to a narrowing of the gene pool (Hernandez-Espinosa et al., 2020; Xynias et al., 2020). To date, no comprehensive studies of the genetic pool of Russian spring durum wheat in terms of mineral composition have been conducted, so it is not yet possible to draw a conclusion regarding the landrace superiority

The search for sources of genetic diversity draws attention to the inclusion of wheat relatives in hybridization in an attempt to increase the content of essential micro- and macronutrients in grains. It is known that tetraploid species such as T. dicoccum, T. dicoccoides, T. timopheevii, and hybrid lines derived from them are characterized by significantly higher levels of zinc and iron in grains (Cakmak et al., 2010; Del Coco et al., 2019; Tekin et al., 2022; Leonova et al., 2024). The data on the origin of the seven breeding lines from the Samara Scientific Research Agriculture Institute that formed a separate cluster indicate that tetraploid species T. dicoccum and T. timopheevii were used in their development. A previous microsatellite analysis of early generations of these lines revealed the presence of alien translocations from T. timopheevii in chromosome 6B (Malchikov et al., 2015). However, further research is needed to establish a link between the presence of alien insertions in the genome and their effect on the content of specific elements

## Conclusion

The assessment of micro- and macronutrient and toxic metal content in grains of spring durum wheat varieties conducted in this study revealed significant variation in most elements depending on genotype and environmental conditions. The presence of significant positive correlations Fe/Mn, Fe/Zn, and Zn/Mn indicates the possibility of selecting genotypes based on multiple micronutrients simultaneously. The analysis of the results suggests that Russian varieties and breeding lines exhibit moderate genetic diversity; however, the observed range of trait variation allows for the identification of samples that can be used to improve the mineral composition of grain. It was shown that Cd content in grains of all studied samples, except for five foreign varieties, does not exceed the maximum permissible concentrations (MPCs). To determine the cause of the elevated Zn, Pb, and Cr contents exceeding the MPCs in some samples, additional data on the concentration of these metals in the soil of the experimental plots are required.

## Conflict of interest

The authors declare no conflict of interest.
